# The Association of Tryptophan and Its Metabolites With Incident Hip Fractures, Mortality, and Prevalent Frailty in Older Adults: The Cardiovascular Health Study

**DOI:** 10.1002/jbm4.10801

**Published:** 2023-08-18

**Authors:** Laura Carbone, Petra Bůžková, Howard A Fink, John A Robbins, Joshua I Barzilay, Rachel E Elam, Carlos Isales

**Affiliations:** ^1^ Division of Rheumatology, Department of Medicine Augusta University Augusta GA USA; ^2^ Charlie Norwood Veterans Affairs Medical Center Veterans Affairs Health Care System Augusta GA USA; ^3^ Department of Biostatistics University of Washington Seattle WA USA; ^4^ Geriatric Research Education and Clinical Center Veterans Affairs Health Care System Minneapolis MN USA; ^5^ Department of Medicine University of California Davis Davis CA USA; ^6^ Division of Endocrinology, Kaiser Permanente of Georgia Emory University School of Medicine Atlanta GA USA; ^7^ Division of Endocrinology, Department of Medicine Augusta University Augusta GA USA

**Keywords:** AGING, FRACTURE PREVENTION, GENERAL POPULATION STUDIES, OSTEOPOROSIS, STATISTICAL METHODS

## Abstract

Amino acids are the building blocks of proteins, and sufficient protein intake is important for skeletal health. We utilized stored serum from the Cardiovascular Health Study in 1992–1993 to examine the relationship between levels of the essential amino acid tryptophan (trp) and its oxidized and nonoxidized metabolites to risk for incident hip fractures and mortality over 12 years of follow‐up. We included 131 persons who sustained a hip fracture during this time period and 131 without a hip fracture over these same 12 years of follow‐up; 58% female and 95% White. Weighted multivariable Cox hazards models were used to estimate the hazard ratios (HR) and 95% confidence intervals (CI) of incident hip fracture associated with a one standard deviation (SD) higher trp or its metabolites exposure. Relative risk regression was used to evaluate the cross‐sectional association of trp and its metabolites with frailty. Higher serum levels of trp were significantly associated with lower risk of incident hip fractures (HR = 0.75 per SD of trp (95% CI 0.57–0.99) but were not significantly associated with mortality or frailty status by Freid's frailty index. There were no statistically significant associations between any of the oxidized or nonoxidized products of trp with incident hip fractures (*p* ≥ 0.64), mortality (*p* ≥ 0.20), or cross‐sectional frailty status (*p* ≥ 0.13) after multiple testing adjustment. Randomized clinical trials examining whether increasing trp intake is beneficial for osteoporosis are needed. © 2023 The Authors. *JBMR Plus* published by Wiley Periodicals LLC on behalf of American Society for Bone and Mineral Research. This article has been contributed to by U.S. Government employees and their work is in the public domain in the USA.

## Introduction

Hip fractures and frailty are two fundamental problems of aging.^(^ ^1^, ^2^ ^)^ There is growing interest on whether optimizing protein intake might be beneficial for both osteoporosis and frailty,^(^ ^3^, ^4^, ^5^, ^6^ ^)^ given the prevalence of dietary protein insufficiency with age.^(^ ^7^ ^)^ However, to date, there are no established recommendations for levels of protein intake to prevent osteoporosis or frailty. This stems in part from the fact that protein has both benefits and risks to the skeleton. Beneficial effects may be attributable to increasing insulin‐like growth factor^(^ ^8^ ^)^ and potential negative effects may be mediated by increases in metabolic acidosis and hypercalciuria.^(^ ^9^ ^)^ Some^(^ ^3^, ^4^, ^5^, ^6^, ^10^, ^11^, ^12^ ^)^ but not all^(^ ^13^, ^14^, ^15^ ^)^ studies have reported beneficial associations of higher protein intakes with fracture risk. The few randomized clinical trials of increasing protein intakes through diet or supplements that have examined changes in bone mineral density (BMD) have also had inconsistent results.^(^ ^16^, ^17^, ^18^, ^19^, ^20^, ^21^, ^22^ ^)^ Several reports have considered the source of protein in their analysis, ie, animal versus plant protein, but here, too, the findings are conflicting.^(^ ^4^, ^23^, ^24^, ^25^ ^)^ The association of specific amino acids with skeletal health is now a focus of study.^(^ ^26^, ^27^, ^28^, ^29^, ^30^ ^)^

Tryptophan (trp) is an essential aromatic amino acid that is a basic building block for neurotransmitters and hormones.^(^ ^31^, ^32^, ^33^ ^)^ In experimental studies in which rats were given trp‐free diets, there were both decreases in bone formation and increases in bone resorption noted.^(^ ^34^ ^)^ Abnormalities in the trp pathway have been identified in metabolomic studies of aging.^(^ ^3^, ^35^ ^)^ However, much of the data on trp and trp metabolites’ effects on bone and muscle come from animal, not human, studies.

Age‐related increases in reactive oxygen species (ROS) likely contribute to physiologic aging by inducing cell damage and altering normal protein function. Increases in ROS have been associated with decreased wingless‐related integration site (Wnt), hedgehog, bone morphogenetic protein (BMP), and extracellular signal relating kinase (ERK) signaling pathways, all of which are important for differentiation of osteoblasts, and may be why ROS negatively impact skeletal health.^(^ ^4^ ^)^ Cytosolic (eg, glutathione synthase) and membrane (eg, CoQ10) associated antioxidant systems mitigate this damage, but antioxidant enzymatic defense systems are impaired with aging.^(^ ^5^ ^)^Trp is particularly vulnerable to oxidative modification through the kynurenine (kyn) pathway.^(^ ^6^, ^7^, ^8^, ^9^ ^)^ Further, in mouse bone marrow stem cells, oxidation of trp leads to loss of its anabolic effect on bone cells.^(^ ^10^ ^)^ The first metabolite in trp degradation is kyn, which in experimental models increases ROS and lipid peroxidation and interferes with immune and stem cell function and osteoblastogenesis.^(^ ^11^, ^12^ ^)^ In a study by members of our group, kyn treatment of mouse and human myoblasts doubled levels of ROS and, in vivo, reduced muscle size and strength and increased muscle lipid peroxidation in young mice.^(^ ^13^ ^)^ Others have reported that feeding young mice increasing kyn concentrations lowers bone volume and increases bone resorption.^(^ ^14^ ^)^

However, some of the metabolites generated by trp degradation are actually pro‐osteogenic rather than pro‐resorptive; moreover, the ultimate effects of the metabolite on bone may depend on the local redox environment.^(^ ^11^ ^)^ Hydroxyanthranilic acid (3‐HAA), a trp metabolite, can either act as an antioxidant or a pro‐oxidant, and whether it has antioxidant or pro‐oxidant actions depends in part on local conditions.^(^ ^15^ ^)^ Picolinic acid, another trp metabolite, has been reported to have anabolic effects on bone in mice.^(^ ^16^ ^)^

Few studies in humans have examined the relationship of trp and its metabolites with osteoporosis, mortality, and frailty.^(^ ^17^, ^18^, ^19^, ^20^, ^21^, ^22^ ^)^ We utilized stored serum and longitudinal cohort data from the Cardiovascular Health Study (CHS) to measure trp and its oxidized and nonoxidized metabolites and determine their association with incident hip fractures and mortality and cross‐sectional associations with frailty status. We hypothesized that the oxidized metabolites of trp would be directly associated and trp and its nonoxidized metabolites would be inversely associated with hip fractures, mortality, and frailty.

## Participants and Methods

### Study participants

We included persons participating in the CHS, a longitudinal cohort of community‐dwelling adults aged 65 years and older.^(^ ^23^, ^36^ ^)^ The original cohort of 5201 CHS participants was enrolled in 1989–1990, and an additional 687 Black men and women were included in 1992–1993. The CHS was approved by institutional review boards at all four of its study sites, and all subjects gave written informed consent to participate. Yearly in‐person clinic examinations, which included blood sampling, were done during years 1989–1999; after this, data, including information on any hospitalizations, medical conditions, and medication use, were collected at visits every 2 years.

Funding was available for serum measurements in 262 participants. We sampled from the original cohort participants who attended the 1992–93 visit (4578) who had not sustained a hip fracture in the previous 3 years of follow‐up (4526) and had complete information on the following covariates: age, race, sex, health status, history of diabetes, education, smoking and alcohol use history, physical activity, alcohol use, estimated glomerular filtration rate (eGFR)‐cystatin, body mass index (BMI), medication use, and had serum available (*n* = 3192). From these, we selected 131 participants without an incident hip fracture during the 12 years after the 1992–93 visit and 131 participants with an incident hip fracture during the 12 years. Further, a race‐ and sex‐stratified sample was selected for each of the two 131 hip fracture status groups, and inverse probability of sampling weights accompanied the data.

### Measurements of trp and trp metabolites

Blood samples were selected from a single time point, the 1992–93 CHS visit. These were collected after a minimum 8‐hour fast, and serum was immediately separated and frozen for long‐term storage at −80°C. Specimens for these analyses were measured using liquid chromatography‐mass spectrometry (LC‐MS). All measurements are reported in fmol/μL.

### Outcomes

#### Hip fractures

Incident hip fractures were first ascertained by participant report and confirmed by review of hospital medical records. To include any hip fractures not reported by the participant, hospital claims data and hospitalization discharge summaries were also reviewed.^(^ ^36^ ^)^ Hip fracture was defined using the International Classification of Diseases, Ninth Revision (ICD‐9), codes (820.xx). Pathological fractures (ICD‐9 code 773.1x) and motor vehicle accidents (E810.xx‐E825.xx) were excluded. Follow‐up for hip fractures was for 12 years after the 1992–93 visit of CHS.

#### Mortality

Information on mortality was collected at the in‐clinic visits and also at 6‐month telephone interviews. Deaths were also ascertained by review of obituaries, medical records. and proxy interviews. Follow‐up for mortality ended at 12 years after the 1992–93 visit of CHS and was 100% complete for this time period.

#### Frailty

The operational definition of frailty originally developed in CHS by Fried (known as the frailty Fried's index) was used to define frailty^(^ ^23^ ^)^ for these analyzes. This included at least three of the following five outcomes: slow walking speed during a 4.5 m walk, muscle weakness, low physical activity, weight loss, and self‐reported exhaustion. Frailty parameters were defined in a sex‐specific manner for walking speed and muscle weakness. Muscle weakness was determined by grip strength, which was measured with a Jamar hydraulic hand dynamometer. The best result of three attempts was taken as the result. Low physical activity was determined by the patient answering “less” to the question, “Are you more, less, or equally active compared to men and women of your age?” Weight loss was defined as unintentional weight loss of >5 kg during the last year or a BMI of <18.5. Self‐reported exhaustion was reported. Prefrail was defined as 1 or 2 of these conditions and not frail when none of these five criteria were met.^(^ ^23^ ^)^ We assessed frailty cross‐sectionally at the 1992–93 CHS visit.

#### Assessment of covariates

Covariate information was obtained from the 1992–93 CHS visit, the same year as the serum samples used in these analyzes. We a priori selected potential covariates that could impact the association of trp/metabolites and hip fractures and/or frailty. We included the following covariates in these analyses: age, BMI (kg/m^2^), sex, race, self‐reported health status (excellent, very good, good, fair, poor), history of diabetes, smoking status (current, former, never), clinic site, highest education level completed (≥12th or <12th grade), current alcohol use (0 to ≤7 drinks/week, >7 drinks/week), and renal function from eGFR from combined creatinine‐cystatin C equation.^(^ ^24^ ^)^ Use of medications was identified by direct examination of the medication bottles. We included medications used to treat or prevent osteoporosis (selective estrogen receptor modulators, estrogens, and bisphosphonates) and medications associated with fracture risk, including oral corticosteroids, loop diuretics, thiazide diuretics, selective serotonin reuptake inhibitors, anticonvulsants, benzodiazepines, sedative/hypnotics, proton pump inhibitors, thiazolidinedione, and thyroid medications.

#### Dietary intakes

We included dietary intakes of trp, which were collected in 1989–90 using a qualitative, picture‐sort food frequency questionnaire (FFQ). In a substudy validation of the CHS FFQ including 47 female and 49 males aged 66 to 100 years from the CHS, estimates of mean nutrient intakes from the picture‐sort FFQ used in CHS were comparable to estimates based on 24‐hour recalls, and correlations with reference data were similar to those reported in the literature for conventionally administered FFQ.^(^ ^25^, ^26^ ^)^

### Statistical analyses

Weighted multivariable Cox hazards models were used to estimate the hazard ratios (HR) and 95% confidence intervals (CI) of incident hip fracture associated with a one standard deviation (SD) higher trp or metabolite exposure. SDs were as follows: trp 2446; kyn 108; kyn/trp 0.02; kynurenic acid 5; xanthurenic acid 4; quinolinic acid 25; 3‐HAA 4; picolinic acid 91; oxidized metabolites 113; nonoxidized metabolites 93; and oxidized/nonoxidized metabolites 0.7.

The outcome data of incident hip fracture during follow‐up are used in a time‐to‐event analysis subject to right‐hand censoring for death; this design is not a case–control design. For the purposes of these analyses, data were censored at loss to follow‐up, death, or after 12 years of follow‐up after the 1992–93 CHS visit. Robust standard errors were used due to weighting of the participants. Cross‐sectionally, weighted Poisson regression models with robust standard errors were used to estimate the relative risk of frailty associated with a standard deviation higher exposure. The frailty index included categories of frailty, prefrail, and not frail, which were converted to binary indexes. We used nested models adjusting for factors as follows: age and sex adjusted, minimally adjusted (age, sex, race, clinic site), and fully adjusted: age, BMI (kg/m^2^), sex, race, clinic site, self‐reported health status (excellent, very good, good, fair, and poor), history of diabetes, smoking status (current, former, never), highest education level completed (≥12th or <12th grade), renal function, current alcohol use (0 to ≤7 drinks/week, >7 drinks/week), and medication use. We determined Pearson correlation coefficients between dietary intakes of trp and serum trp levels.

The primary exposure was trp; trp metabolites were secondary exposures. We also calculated a ratio of a composite of oxidized metabolites over a composite of nonoxidized metabolites as a secondary exposure. For secondary exposures, in addition to the original *p* values, a multiple testing approach based on false discovery rate^(^ ^27^ ^)^ was done to calculate adjusted *p* values.

Three individuals had kynurenic acid with values >100, which were considered outliners and eliminated from the analyses. Seventy‐three individuals had values of 3‐hydroxyanthranilic acid (3‐HAA) below the limit of detection; these were then coded as 0.02, which was the lowest non‐zero value in all measurements.

## Results

Table 1 depicts the distribution of values for trp and its metabolites. The intra‐assay and interassay coefficients of variation (CVs) for each biomarker are shown in Table 2. The mean age of the study population was 75 years, 42% were men, and the majority were White. The proportion who were frail increased over follow‐up (Table 3). Higher levels of trp were significantly associated with a lower risk for incident hip fracture (HR = 0.75 per SD of trp; 95% CI 0.57–0.99) (Table 4). No metabolite of trp was significantly associated with hip fracture risk (Table 4), and oxidation status of these metabolites was not significantly associated with hip fracture risk (Table 5).

**Table 1:** Distribution of Values of Tryptophan (Trp) and Its Metabolites

Trp and metabolites	*N*	Min	Q1	Median	Q3	Max
Trp	262.00	1439.18	7988.45	9271.61	10694.85	15498.85
Kynurenine (kyn)	262.00	173.32	288.60	349.81	424.00	956.37
Kyn/Trp	262.00	0.02	0.03	0.04	0.04	0.19
Kynurenic acid	259.00	2.52	7.64	9.46	12.41	42.43
Xanthurenic acid	262.00	0.00	0.29	2.33	5.08	29.89
Quinolinic acid	262.00	0.00	13.71	21.69	37.23	386.71
3‐hydroxyanthranilic acid (3‐HAA)	262.00	0.02	0.02	3.71	7.47	26.43
Picolinic acid	262.00	103.01	307.23	350.61	408.33	539.76

**Table 2:** Intra‐assay and Interassay Coefficients of Variation (CV) for Serum Measurements of Tryptophan (Trp) Metabolites

Coefficients of Variation (CV)	L‐kynurenine	Picolinic Acid	3 Hydroxyanthranilic acid (3‐HAA)	Kynurenic acid	Quinolinic acid	Serotonin	Xanthurenic Acid
CV intra‐assay	3.8%	7.6%	26.1%	10.1%	7.0%	10.6%	13.0%
CV interassay	7.5%	12.9%	27.4%	19.2%	7.0%	8.5%	13.3%

**Table 3:** Weighted Demographic and Clinical Characteristics of Study Population^a^

	Mean ± SD (OR %)		
Characteristics	All mean	Before hip fracture	During follow‐up
Age (years)	75.0 (4.7)	74.9 (4.6)	76.5 (5.1)
Body mass index (kg/m^2^)	25.8 (4.3)	25.8 (4.3)	25.5 (4.0)
Sex (%)			
Male	42	43	28
Female	58	57	72
Race (%)			
Black	5	5	2
White	95	95	98
Health status (%)			
Excellent	4.6	4.7	2.9
Very good	35.7	36.6	27.7
Good	47	46.9	48.3
Fair	12.5	11.8	18.7
Poor	0.2	0	2.4
Diabetes (%)	13.7	14	11.6
Smoking history (%)			
Current	7.5	7.7	6.3
Former	48.2	49.3	37.1
Never	44.3	43	56.7
Clinic site (%)			
Bowman Gray, NC	24.6	23.8	32.8
Hagerstown, MD	25.3	24.7	30.4
Davis, CA	24.3	25.1	16.9
Pittsburgh, PA	25.8	26.4	19.9
Education (%)			
<12th grade	45	45	47
>12th grade	55	55	53
Renal function^b^	72.8 (19.2)	72.8 (19.4)	72.8 (16.5)
Current alcohol use (%) (drinks/week)			
0–≤7	88	88	95
>7	12	12	5
Medication use (%)			
Osteoporosis medication use^c^	3.6	3.6	3.2
Bone active medication use^d^	29.5	28.4	40.2
Thyroid medication use^e^	8.8	8.4	12.6
Frailty status (%)^f^			
Not frail	48.7	50.7	28.6
Pre‐frail	43.6	42.6	54.3
Frail	7.7	6.8	17.1

^a^ Based on cumulative incidence of hip fractures.

^b^ Estimated glomerular filtration rate calculated from serum creatinine and cystatin C.

^c^ Defined as use of selective estrogen receptor modifiers (SERMs), estrogens, or bisphosphonates.

^d^ Defined as use of loop diuretics, thiazide diuretics, selective serotonin reuptake inhibitors, anticonvulsants, benzodiazepines, sedatives/hypnotics, proton pump inhibitors, thiazolidinediones.

^e^ Defined as use of thyroid medications.

^f^ Freid's frailty index.

**Table 4:** Association of Tryptophan (Trp) and Its Metabolites With Hip Fractures

Predictors	Age and sex adjusted	*p* Value	Minimally adjusted, HR (95% CI)^a^	*p* Value	Fully adjusted,^b^ HR (95%CI)	*p* Value
Primary predictor						
Trp^d^	0.79 (0.61–1.02)	0.07	0.79 (0.61–1.02)	0.07	0.75 (0.57–0.99)	0.04
Secondary predictors						
Kynurenine (kyn)^d^	0.79 (0.6–1.03)	0.87	0.77 (0.57–1.02)	0.81^c^	0.63 (0.41–0.96)	0.64^c^
Kyn/Trp^d^	0.96 (0.76–1.21)	1.00^c^	0.96 (0.76–1.21)	1.00^c^	0.98(0.74‐1.29)	1.00^c^
Kynurenic acid^d^	0.86 (0.66–1.12)	1.00^c^	0.85 (0.66–1.09)	1.00^c^	0.84 (0.62–1.31)	1.00^c^
Xanthurenic acid^d^	0.99 (0.73–1.33)	1.00^c^	0.97 (0.71–1.33)	1.00^c^	1.09 (0.77–1.54)	1.00^c^
Quinolinic acid^d^	1.08 (0.84–1.4)	1.00^c^	1.04 (0.78–1.38)	1.00^c^	1.06 (0.75–1.49)	1.00^c^
3‐Hydroxyanthranilic acid (3‐HAA)^d^	1.33 (1.08–1.63)	0.27	1.28 (1.04–1.57)	0.81^c^	1.25 (1.00–1.55)	0.64^c^
Picolinic acid^d^	1.08 (0.85–1.38)	1.00^c^	1.04 (0.81–1.33)	1.00^c^	1.02 (0.79–1.34)	1.00^c^

^a^ Adjusted for age, sex, race, and clinic site.

^b^ Adjusted for age, body mass index (kg/m^2^), sex, race, clinic site, self‐reported health status (excellent, very good, good, fair, poor), history of diabetes, smoking status (current, former, never), clinic site, highest education level completed (≥12th or <12th grade), renal function, current alcohol use (0 to ≤7 drinks/week, >7 drinks/week), and medication use.

^c^ Adjusted for multiple testing.

^d^ SD: trp 2446; kyn 108; kyn/trp 0.02; kynurenic acid 5; xanthurenic acid 4; quinolinic acid 25; 3‐HAA 4; picolinic acid 91.

**Table 5:** Association of Oxidation Status of Tryptophan (Trp) Metabolites With Hip Fractures

	Age and sex adjusted	*p* Value	Minimally adjusted, HR (95% CI)^a^	*p* Value	Fully adjusted,^b^ HR (95%CI)^a^	*p* Value
Oxidized metabolites (Kyn, kynurenic acid, xanthurenic acid, quinolinic acid)	0.83 (0.63–1.09)	1	0.81 (0.61–1.09)	1	0.70 (0.46–1.07)	0.99
Nonoxidized metabolites (3‐HAA, picolinic acid)	1.09 (0.85–1.4)	1	1.05 (0.81–1.34)	1	1.03 (0.79,1.35)	1
Ratio of oxidized/nonoxidized metabolites (Kyn, kynurenic acid, xanthurenic acid, quinolinic acid/(3‐HAA, picolinic acid)	0.78 (0.59–1.02)	0.87	0.77 (0.57–1.03)	0.81	0.74 (0.51–1.06)	0.85

^a^ Adjusted for age, sex, race, and clinic site.

^b^ Adjusted for age, body mass index (kg/m^2^), sex, race, clinic site, self‐reported health status (excellent, very good, good, fair, poor), history of diabetes, smoking status (current, former, never), highest education level completed (≥12th or <12th grade), renal function, current alcohol use (0 to ≤7 drinks/week, >7 drinks/week), and medication use.

^c^ Adjusted for multiple testing.

^d^ SD: oxidized metabolites 113, nonoxidized metabolites 93, and oxidized/nonoxidized metabolites 0.7.

Associations of trp or its metabolites with mortality were not statistically significant in multivariable models (Table 6). There was no significant association of trp or its metabolites with Freid's frailty status, including examination of references of frail versus non‐frail or pre‐frail, pre‐frail versus non‐frail with frail persons excluded, and frail or pre‐frail versus non‐frail (*p* ≥ 0.13 for all) (data not shown). Serum levels of trp were not significantly associated with dietary intakes (*R* = −0.118).

**Table 6:** Association of Tryptophan (Trp) and Its Metabolites With Mortality

Predictors	Age and sex adjusted	*p* Value	Minimally adjusted,^a^ HR (95% CI)	*p* Value	Fully adjusted,^b^ HR (95% CI)	*p* Value
Primary predictor						
Trp	0.85 (.069–1.05)	0.14	0.94 (0.79–1.12)	0.51	0.87 (0.74–1.03)	0.11
Secondary predictors						
Kynurenine (kyn)	1.05 (0.85–1.29)	1	1.16 (0.99–1.37)	0.35^c^	1.04 (0.86–1.27)	1.00^c^
Kyn/Trp	1.07 (0.91–1.25)	1	1.04 (0.89–1.22)	1.00^c^	1.10 (0.95–1.27)	1.00^c^
Kynurenic acid	1.18 (0.96–1.45)	0.68	1.17 (1.01–1.36)	0.23^c^	1.01 (0.87–1.17)	1.00^c^
Xanthurenic acid	1.22 (0.98–1.5)	0.62	1.14 (0.96–1.34)	1.00^c^	1.11 (0.95–1.30)	1.00^c^
Quinolinic acid	1.25 (1.12–1.4)	<0.01	1.23 (1.14–1.37)	0.01^c^	1.17 (1.01–1.35)	0.36^c^
3‐Hydroxyanthranilic acid (3‐HAA)	1.19 (0.98–1.45)	0.62	1.17 (1.00–1.35)	0.27^c^	0.95 (0.81–1.11)	1.00^c^
Picolinic acid	1.28 (1.02–1.62)	0.48	1.27 (1.08–1.49)	0.05^c^	1.21 (1.04–1.41)	0.29^c^

^a^ Adjusted for age, sex, race, and clinic site.

^b^ Adjusted for age, body mass index (kg/m^2^), sex, race, clinic site, self‐reported health status (excellent, very good, good, fair, and poor), history of diabetes, smoking status (current, former, never), highest education level completed (≥12th or <12th grade), renal function, current alcohol use (0 drinks/week to ≤7 drinks/week, >7 drinks/week), and medication use.

^c^ Adjusted for multiple testing.

There were only 10 persons taking any medication for osteoporosis. Sensitivity analyses, excluding these 10 persons, yielded similar findings to the results for the whole population (data not shown).

## Discussion

Higher levels of tryptophan, but none of its oxidized or nonoxidized metabolites, were significantly associated with a lower risk of incident hip fractures in men and women in the Cardiovascular Health Study.

Our findings that trp was inversely associated with hip fractures are supportive of our hypothesis and are consistent with prior reports of a benefit of trp on skeletal health. In a study including men with osteoporosis, trp levels were significantly positively associated with BMD at both the lumbar spine and hip.^(^ ^28^ ^)^ In a study including 33 patients, those with osteoporosis had decreased levels of trp.^(^ ^17^ ^)^ In the Hordaland Health Study, plasma trp levels were significantly positively correlated with BMD.^(^ ^29^ ^)^ Our findings showing a beneficial association of trp with decreased hip fracture risk in a geographically diverse US population extend those of a prior study in which higher serum levels of trp were associated with a lower risk of major osteoporotic fractures in a Chinese population in Hong Kong.^(^ ^30^ ^)^

That the oxidized metabolites of trp, including kyn, kynurenic acid, and xanthurenic acid, were not significantly related to risk for hip fractures in CHS was counter to our hypothesis but in agreement with findings from the Hordaland Health Study.^(^ ^31^ ^)^ Our work extends findings from this report^(^ ^31^ ^)^ to include that neither the oxidized metabolite quinolinic acid nor the nonoxidized metabolites 3‐HAA and picolinic acid are significantly associated with hip fracture risk. In contrast, in a small series of 39 patients with and without osteoporosis, those with osteoporosis had significantly lower levels of 3‐HAA and higher levels of anthranilic acid when compared with the healthy controls,^(^ ^19^ ^)^ although this small series did not report fracture information.

The mechanisms by which serum levels of trp but not its metabolites were associated with hip fracture risk in older men and women are not readily apparent. One possible cause for this discrepancy is that there are mediators, not controlled for in these analyses, that are associated with both the rate of trp metabolism and hip fracture risk. Candidate mediators, where current literature supports both an association with concentration of serum trp and hip fracture risk, include circulating interferon gamma cytokine levels and amount of ingested antioxidants. Trp is broken down to kynurenine primarily through three enzymes, tryptophan 2, 3‐dioxygenase (TDO) located in the liver, and indoleamine 2, 3‐dioxygenase (IDO‐1)^(^ ^14^, ^32^ ^)^ expressed in lymphoid tissue and immune cells among others.^(^ ^14^ ^)^ IDO‐1 is inducible by inflammatory cytokines such as interferon gamma.^(^ ^14^ ^)^ Therefore, higher concentrations of trp would be expected in lower interferon gamma signaling. In women with postmenopausal osteoporosis, compared with healthy controls, less basal secretion of interferon gamma by CD4+ cells was reported by Breuil and colleagues.^(^ ^32^ ^)^ Interferon gamma may promote bone loss through upregulation of RANKL and TNF‐alpha.^(^ ^33^ ^)^ In addition to inflammation, IDO‐1 activity is also modulated by diet. Trp ingested as part of a meal appears to have biological effects that are qualitatively different from just taking a trp supplement because of the co‐ingestion of other amino acids and antioxidants present in a meal.^(^ ^34^ ^)^ Foods rich in antioxidants increase circulating trp and are protective against hip fracture.^(^ ^37^ ^)^ Low levels of antioxidants are associated with osteoporosis in postmenopausal women and increased reactive oxygen species are negatively associated with BMD in adults, suggesting an association of more ingested antioxidants and higher BMD.^(^ ^38^, ^39^ ^)^ Notably, our dietary information on trp, which suggested no correlation between diet and serum levels, was from several years before the serum measurements.

In CHS, there was a nonsignificant trend for trp to be inversely associated with mortality. Though it is not possible to draw conclusions from this finding, in a study including almost 2000 patients with coronary artery disease, higher plasma levels of trp were associated with less mortality.^(^ ^40^ ^)^ Our finding that kyn was not associated with mortality is in contrast with this study, which reported that higher levels of kyn were significantly associated with mortality.^(^ ^41^ ^)^ Unlike our work, this study assessed urinary and not serum kyn levels and participants were confined to those with suspected coronary artery disease.^(^ ^41^ ^)^

In previous animal studies from some members of our group, we reported that trp supplementation increased skeletal muscle IGF‐1 and increased expression of MyoD, myogenin, and myosin heavy chain.^(^ ^42^ ^)^ However, with few individuals with prevalent frailty in our analyses, the fully adjusted risk of trp with frailty was not statistically significant. Our findings in CHS that kyn/trp ratios were not significantly associated with frailty are consistent with a study including 180 older adults, in which kyn/trp ratios were not significantly associated with frailty.^(^ ^20^ ^)^ However, in a cohort of Spanish elderly individuals age 65 and older, significantly higher kyn/trp ratios were reported in those with frailty.^(^ ^21^ ^)^ In contrast with our findings of no association of trp levels with frailty, in older Black men in the Health, Aging and Body Composition (Health ABC) study, lower plasma levels of trp were significantly associated with frailty.^(^ ^43^ ^)^ This study differs from ours as we had few Black men.^(^ ^43^ ^)^ Also differing from our findings in CHS, in 73 participants undergoing a geriatric assessment, higher serum kyn levels were associated with frailty.^(^ ^44^ ^)^ Participants in this geriatric assessment had an average age of 69 years, slightly younger than our CHS participants, whose mean age was 75 years; however, the reasons for these discrepant findings are not entirely clear. In CHS, none of the other trp metabolites measured were associated in frailty. In contrast, in 85 participants in the Nepean Osteoporosis and Frailty Study, kynurenic acid was associated with a lower risk for frailty and quinolinic acid with a higher likelihood for frailty^(^ ^45^ ^)^; however, a history of a fracture within 3 months was an exclusion criteria for this study, whereas in our CHS cohort, by study design, 50% had sustained a hip fracture.

Our study has numerous strengths. We selected participants from within a cohort study in which hip fracture and frailty outcomes have been well characterized. We were able to measure several important trp metabolites, including both oxidized and nonoxidized metabolites. Finally, we examined a number of outcomes important to the aging population, including hip fractures, mortality, and frailty.

The report also has several limitations. We only evaluated associations with hip and not other fracture sites. Our cohort had a limited number of Blacks and no other ethnic minorities. Further, we were only able to include serum measurements from 131 persons with and 131 persons without a hip fracture due to budgetary constraints and thus may have incurred selection bias. We did not examine changes in frailty status over time because data on changes in frailty status was missing for the majority of the individuals included in these analyses. Biomarkers were measured only from a single time point. We did not include measurements of BMD, as dual‐energy X‐ray absorptiometry (DXA) measurements were only performed in 73 of the participants included in these analyses. The CVs in our laboratory were high for some biomarkers, in particular, for 3‐HAA. Further, we did not measure levels of indoleamine‐pyrrole 2, 3‐dioxygenase (IDO), which is a key regulator of trp metabolism.^(^ ^46^ ^)^ The serum measurements of trp were done several years after the dietary information was collected in CHS and diet may have changed over this time.

In summary, trp, but not its metabolites, was significantly inversely associated with hip fractures. The challenge will be to identify how to determine optimal intakes of trp for fracture prevention.

## Disclosures

No authors have any disclosures related to this work.

### Peer Review

The peer review history for this article is available at https://www.webofscience.com/api/gateway/wos/peer‐review/10.1002/jbm4.10801.
